# Effectiveness of a Multidisciplinary Treatment Program for Severe Obesity in Adults Based on the Clinically Significant Weight Loss

**DOI:** 10.3390/jfmk10020225

**Published:** 2025-06-11

**Authors:** Greice Westphal-Nardo, Angélica Sbrolini Marques Mincache, Paulo César Franzini, Mara Jane Pascoini dos Santos, Gisele Nicchio Rocha, Ieda Carla Candido, Andrea Herrera-Santelices, Felipe Merchan Ferraz Grizzo, Jean-Philippe Chaput, Nelson Nardo Junior

**Affiliations:** 1 Department of Physical Education, Associate Graduate Program in Physical Education UEM/UEL, Health Sciences Center, State University of Maringa, Maringa 87020-900, Parana, Brazil; greicewnardo@uepg.br (G.W.-N.); prof.angelicamincache@gmail.com (A.S.M.M.); nemo.organizacao@gmail.com (P.C.F.); marapascoini2@gmail.com (M.J.P.d.S.); giselebuzzo04@gmail.com (G.N.R.); iedacarlacandido@hotmail.com (I.C.C.); nnjunior@uem.br (N.N.J.); 2 Healthy Active Living and Obesity Research Group, Children’s Hospital of Eastern Ontario Research Institute, Ottawa, ON K1H 8L1, Canada; jpchaput@cheo.on.ca; 3 Center for Multiprofessional Studies on Obesity—NEMO/HUM/UEM, University Hospital of Maringa, State University of Maringa, Maringa 87083-240, Parana, Brazil; fmfgrizzo@gmail.com; 4 Department of Physical Education, Biological and Health Sciences Sector, State University of Ponta Grossa, Ponta Grossa 84030-000, Parana, Brazil; 5 Departamento de Ciencias Pre-Clínicas, Facultad de Medicina, Universidad Católica del Maule, Talca 3460000, Región del Maule, Chile

**Keywords:** obesity, severe obesity, multidisciplinary treatment, weight loss, effectiveness, Brazil

## Abstract

**Background:** Obesity is a chronic and complex disease; by its nature, it represents an enormous challenge to be solved and managed. For that matter, several guidelines have been published, but there is still a long way to go until concrete scaled results can be presented. Adults with obesity, and especially severe obesity, need to have access to treatment programs, but they are not available for the vast majority of the population. **Objective:** The aim of this study is to evaluate the effectiveness of a multidisciplinary treatment program for obesity (MTPO) offered to adults (ages 18 to 50 years old) with a BMI over 30 kg/m^2^. **Methods:** Participants were invited through media ads, resulting in 404 participants for the first phase of that study, from whom the risk profile was assessed. After that, 180 participants (82.8% with severe obesity) concluded the MTPO, which consisted of 48 sessions of exercises and the same number of professional orientations about a healthy lifestyle, including the importance of being physically active, how to improve their eating habits, and how to control their emotions. **Results:** For the analysis of results, participants were grouped according to their weight loss in terciles, with the first, tercile presenting an average weight loss of 7.6%, which is considered clinically significant. In the same way, the average percental variations were even higher in this group for body fat (12.7%) and the lean mass to fat mass ratio (LM/FM), which increased by 14.3%. The homeostatic model assessment for insulin resistance, HOMA-IR, was around 3 times the variation of body mass, whereas the triglycerides (TG) and the hemoglobin A1C (H1Ac) were around twice that rate. **Conclusions:** These results made clear the effectiveness of the MTPO, which needs to be tested in public health services.

## 1. Introduction

Over the past three decades, obesity has emerged as a pervasive global health crisis [1]. Once considered primarily a concern in high-income nations, the most significant rise and highest prevalence are now observed in low- and middle-income countries [2]. Data from Brazil’s VIGITEL surveillance system, operational since 2006, indicate that over 60% of Brazilian adults were overweight in 2023. The prevalence is higher among men (63.4%) than women (59.6%), and a worsening trend in both overweight and obesity has been consistently observed in adults since the system’s inception [3]. The adoption of unhealthy lifestyle habits, including low levels of physical activity and poor dietary choices, has led to numerous population-level consequences, most notably the increasing prevalence of obesity [4].

Despite the establishment of national health improvement targets, such as Brazil’s Strategic Action Plan to Combat Chronic Noncommunicable Diseases (2011–2022 and 2021–2030), favorable progress has not materialized. In fact, the trend has moved in the opposite direction. The adult obesity prevalence, which was 15.1% in 2010 and was targeted for stabilization by 2022, rose to 18.9% in 2015, 20.3% in 2019, and, according to the most recent 2023 data, now stands at 24.3% [5].

Although global recognition of rising obesity rates and their profound health implications, as well as efforts by authorities to set and monitor targets, consistent progress remains elusive [2]. This should come as no surprise, given that most efforts have historically focused on persons living with obesity, while the societal norms and conditions shaping lifestyles have received comparatively little attention [6].

The management of obesity is notably complex and has historically received insufficient attention [7,8]. It is important to emphasize that significant challenges remain in establishing objective criteria for diagnosis and clinical decision-making, including prioritization of therapeutic interventions and public health strategies. These were highlighted by The Lancet Diabetes & Endocrinology Commission on Clinical Obesity [9].

Given that obesity is a complex, chronic, and multifactorial disease, initiating treatment in its earlier stages is crucial, especially as weight-related alterations are increasingly prevalent in adults [10,11]. There is a clear need to develop comprehensive strategies and define measurable goals for behavioral change programs aimed at improving health and quality of life. Ideally, these should take the form of multidisciplinary intervention programs (MIPs), encompassing physical exercise, nutritional and psychological counseling, and consistent clinical follow-up [12,13,14]. Evidence supports the effectiveness of MIPs, even among patients with severe obesity [15,16,17].

However, there are still several unresolved issues related to implementing these intervention models in public health settings [18,19,20].

One of the most urgent questions concerns the effectiveness of these MIPs when applied in real-world environments, such as public health services, where conditions often differ substantially from those of randomized controlled trials (RCTs) [21,22]. In this context, pragmatic clinical trials (PCTs) are the most appropriate, as their findings can be translated to both public and private healthcare settings [23,24]. Although this issue remains largely unresolved, it has at least been recognized by some researchers [25,26]. While relatively few PCTs have been conducted in this field, their number is increasing across various countries [27,28].

These studies reinforce the need to use real-world settings, such as health units, sports centers, public parks, or even stadiums, to develop programs that address real problems. In this case, success depends on the sustainability of new behaviors; in other words, the promotion of a healthy lifestyle [29].

The purpose of this study is to verify the effectiveness of an MIP in promoting clinically significant weight loss (defined as 5% or more) in adults with obesity classes I to IV. Notably, the majority (82%) of the participants presented severe obesity [30].

## 2. Materials and Methods

### 2.1. Study Design

This is a pragmatic clinical trial of intervention, carried out and described based on the CONSORT 2010 framework [31]. Pragmatic clinical trials are very important because they seek to answer relevant questions that are applicable to everyday clinical practice. In PCT, the external validity is maximized by having few exclusion criteria and by allowing flexibility in the interpretation of the intervention and management decisions, since its goal is to test interventions, devices, or therapeutic resources that could be applied in real-world settings [32]. Therefore, they are the cornerstone of the comparative effectiveness agenda [33].

This study is part of the integrated project entitled “Effectiveness of a multiprofessional program in the evaluation of cardiometabolic risk factors and treatment of abdominal obesity in two municipalities in northwestern Parana”, which was funded by the Fundação Araucária in the call PPSUS/2016. It had an average score of 4.2 using the PRECIS-2 tool, which is a scale whose maximum score is 5. Therefore, indicating a high degree of pragmatism [34].

### 2.2. Participants

Participants were invited to voluntarily participate in the research based on disclosure in the local media (television, radio, and newspaper) and electronic media (website, institutional e-mail, and Facebook). The following inclusion criteria were adopted: (1) age between 18 and 50 years; (2) being overweight or obese; (3) residing in Maringá or Paranavaí, both in Parana state, Brazil; (4) being available to fully participate in the interventions; (5) not having undergone bariatric surgery; (6) not undergoing any other treatment for obesity (therapies, medications, regular physical exercises); and (7) signing the Free and Informed Consent Form.

Those interested in participating in the Multidisciplinary Treatment Program of Obesity previously went through Phase 1 of this study, Pre-inclusion Assessment, divided into Step 1, in which the eligibility conditions to participate in this study were verified when 774 people were in one of the 14 initial pre-inclusion meetings held between December 2017 and March 2018 and between June and July 2019 in the municipality of Maringá held at the Regional University Hospital of Maringá (HUM). And between December 2019 and January 2020 and February 2022 in Paranavaí at the Regional Meeting Centre, and once more in August 2022 in the municipality of Maringá held at HUM, where they responded to an anamnesis to collect data about their socioeconomic and health status and performed a preliminary assessment to measure body mass, height, BMI, waist circumference (WC), and blood pressure (BP). More details can be found in Westphal-Nardo et al. [29].

**Figure 1:**
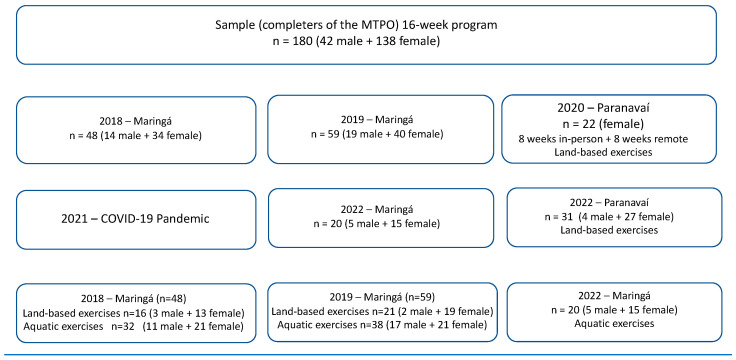
Flowchart of MTPO procedures and exercises.

The phase 2 of this study, called the Multidisciplinary Treatment Program of Obesity, can be classified as an intensive intervention in which the participants enter the treatment program with physical exercise interventions and theoretical classes of healthy eating, psychology, and physical activity to guide and help the participants make changes in their lifestyle. A total of 404 participants, 85 males and 319 females, were considered eligible to participate in the MTPO for 16 weeks, as shown in the flowchart (Figure 1). From those numbers, 166 participants withdrew: 12 due to pregnancy, 46 due to work schedule, 51 due to family or emotional problems (reports of anxiety, depression, and lack of willpower), 33 due to lack of transportation, and 24 with scheduling problems, and the other 58 had no time to be involved in the program as required, and for that reason they did not start it.

A total of 180 participants completed the 16-week MTPO: 42 males and 138 females. In Maringá, 48 participants (14 males and 34 females) concluded in 2018, subdivided into 32 participants in the aquatic exercise (11 males and 21 females) and 16 participants for the terrestrial exercise (3 males and 13 females). In 2019, 59 participants completed the MTPO (19 males and 40 females), subdivided into 38 participants for aquatic exercise (17 males and 21 females) and 21 participants for land exercise (2 males and 19 females). And in 2020, in Paranavaí, with theoretical interventions remotely and with land exercise due to the COVID-19 pandemic, 22 female participants completed the MTPO. And to finish in 2022 in Paranavaí, 31 participants completed the MTPO with land exercise (4 males and 27 females), and in Maringá, 20 participants completed it with aquatic exercise (5 males and 15 females) (Figure 1).

The MTPO’s exercise modalities were established based on facility accessibility during the intervention schedule. Participants in Maringá had access to both land-based and aquatic exercises (water aerobics), whereas those in Paranavaí were limited to land-based exercises due to swimming pool unavailability. Land-based exercise sessions occurred simultaneously. For logistical efficiency, the land-based group was divided, dedicating approximately 30 min to weight training and another 30 min to aerobic exercises. All land-based activities maintained a moderate intensity, targeting 40–59% of the individual’s RHR or a modified Borg RPE of 5–6. Aquatic exercise groups adhered to identical frequency, intensity, and duration parameters. Their water-based routine comprised a 5 min warm-up with escalating intensity, followed by a 50 min conditioning phase, and concluded with a cool-down session that incorporated stretching, deep breathing, and relaxation techniques [35,36].

For the control group (CG), 30 participants were evaluated (11 males and 19 females), and after 16 weeks, they were reassessed. Participants who were unable to attend (have no schedule) for the MTPO or those who withdrew from the intervention within the first month were given the opportunity for reassessment, since they did not engage in any other training or weight loss program.

### 2.3. Ethical Approval

This study has obtained approval from the Research Ethics Committee in accordance with Resolutions 466/2012 and 510/2016 under the registration number CAAE: 56721016.7.1001.0104, approved under n^o^ 2.655.268. Additionally, it has been registered with the Brazilian Registry of Clinical Trials (REBEC) under the registration number RBR2yzs76. Furthermore, this study adheres to the ethical principles outlined in the Helsinki Declaration for medical research involving human subjects.

### 2.4. Statistical Analysis

For the data analyses, a Microsoft Excel 2020 spreadsheet was used, and the statistical tests were performed using the SPSS statistical package version 20.0 [37]. To analyze data distribution, the Shapiro–Wilk test was used to verify normality when the sample was less than 50. For samples larger than 50, the Kolmogorov–Smirnov test was adopted. The data that did not present a normal distribution were normalized by a logarithmic function when necessary; if the majority of the sample presented parametric data, they were presented using descriptive statistics by mean and standard deviation. Data that did not present a normal distribution, considered non-parametric data, were presented by median and interquartile range (percentile). Levene’s test was used to analyze the homogeneity of variances. The Mauchly test was used for variables in which sphericity was violated, and the analyses were adjusted using the Greenhouse–Geisser correction. The unpaired t-test was used to compare variables according to gender and group. To compare variables according to age group and level of obesity, the one-way ANOVA test was used, with Post Hoc Bonferroni for multiple comparisons, to indicate which groups had differences. The Pearson correlation coefficient was used to correlate the variables. The effect size was computed as partial eta-squared values (ηp2; small: ≥0.01, medium: ≥0.06, large: ≥0.14). A significance level of *p* < 0.05 was adopted for all analyses [38,39,40].

## 3. Results

The participants in this study were enrolled in seven MPTO groups offered between 2018 and 2022. They shared common characteristics, including the number of sessions and duration (48 sessions over 16 weeks), as well as the session structure and length. Consequently, they were analyzed collectively.

The dropout rates were relatively high, attributable to various reasons outlined in the methods section, resulting in an average dropout rate of 55.45%, with a minimum of 15.8% and a maximum of 75.9%.

Considering the completers or intervention group (IG) (n = 180), there were 138 females and 42 males. In the control group, there were 11 males and 19 females (n = 30).

Table 1 shows that they have an average age of 39.20 (±7.54) for the CG and 39.27 (±8.82) for the IG. There is no significant difference in age between the groups. The CG had an average body mass of 114.27 (±21.51), and in the IG, it was 109.98 (±23.46), which was a significant difference between the groups at the baseline (*p* < 0.01). The same was observed for BMI, body fat (%), absolute body fat (kg), lean mass/fat mass ratio, waist circumference, abdomen circumference, hip circumference, and waist/height ratio, all with a significant difference between the groups at the baseline (*p* < 0.01).

When comparing the CG and the IG at baseline, Table 1 reveals significant differences in hemodynamic and health-related physical fitness variables, specifically in Diastolic Blood Pressure (DBP) and dynamic lower limb muscular endurance (*p* < 0.01).

Regarding the biochemical parameters, there are also significant differences at baseline in insulin, HOMA-IR, total cholesterol, and LDL-c (low-density lipoprotein cholesterol) (*p* < 0.01). This information suggests that there were notable distinctions between the CG and IG participants in these variables at the outset of this study.

Comparing the baseline values of the indexes derived from the biochemical and anthropometric parameters, Table 1 shows significant differences in the percentile of BMI, MetS-Z WC (metabolic syndrome severity z-score waist circumference), percentile of WC and in TyG-BMI (Triglyceride-Glucose Index) with (*p* < 0.01).

When comparing the different time points (baseline vs. after MTPO or 16 weeks), Table 1 reveals significant differences between the groups in various parameters such as Body Mass: In the CG, there was a significant increase (*p* < 0.01) in body mass, while in the IG, the opposite occurred, with a significant decrease (*p* < 0.01). Concerning BMI, the IG demonstrated a significant reduction (*p* < 0.01), which was not observed in the CG. The same happened for Body Fat (%), Absolute Body Fat (kg), and Neck Circumference with reductions just for IG with (*p* < 0.01). Opposite responses were observed for Waist circumference (cm) and Waist to Height Ratio (cm) with IG showing significant reductions (*p* < 0.01), whereas the CG presented significant increments (*p* < 0.05). The same behavior was observed for the variable lean mass to fat mass ratio for CG and IG, both showing significant increments related to that variable (*p* < 0.01).

Indeed, these findings suggest that the intervention had a significant impact on multiple aspects of body composition and anthropometric measures in the IG when compared to the CG over the course of this study.

Throughout this study, significant differences were observed in hemodynamic and health-related physical fitness parameters between the groups. In the IG, there were notable reductions in both systolic blood pressure (SBP) and DBP, all of which were statistically significant (*p* < 0.01). The same was not observed in the CG.

Furthermore, the IG exhibited substantial improvements in various components of health-related physical fitness. There was a significant increase in static abdominal muscle endurance. Dynamic lower limb muscle endurance also showed significant improvements. Flexibility demonstrated a significant increase, all with (*p* < 0.01). Conversely, the CG experienced a significant reduction (*p* < 0.01) in the distance covered during the 6-Minute Walk Test (6MWT).

These findings underscore the positive impact of the intervention on hemodynamic parameters and multiple components of physical fitness in the IG, whereas the CG primarily showed a decline in 6MWT performance.

Related to the biochemical parameters within IG (pre vs. pos), there were significant reductions in glycemia, insulin, HOMA-IR, HOMA-B, glycated hemoglobin, total cholesterol, LDL-c, VLDL-c (Very-low-density lipoprotein cholesterol), TG, and H1Ac (*p* < 0.01).

Considering the indexes derived from the biochemical and anthropometric parameters within IG, there were significant reductions in AIP (Atherogenic index of plasma), MetS-Z BMI, Percentile BMI, MetS-Z WC, Percentile WC, TyG, TyG-BMI, and TyG-WC (*p* < 0.01).

**Figure 2:**
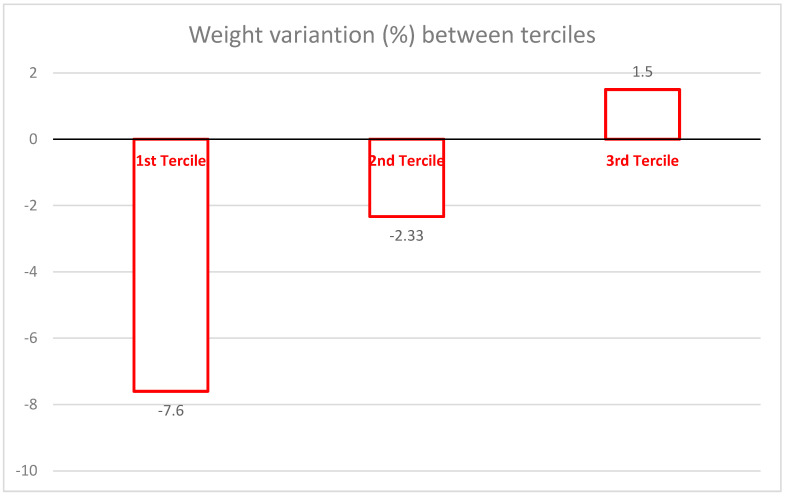
Mean percentage of weight loss according to tercile groups.

Table 2 brings the results stratified by terciles according to the percentual of weight loss. The first tercile is the one whose weight loss was more prominent, with an average percentual weight loss of −7.6%, with the second tercile presenting −2.4%, whereas the third tercile had an average percentual gain of 1.5% (Figure 2). There are significant differences in group 1 (first tercile) compared to the other two groups (*p* < 0.01). The same happened to BMI, Lean Body Mass, Skeletal Muscle Mass, Body Fat %, Absolute Body Fat, LM/FM ratio, WC, Abdominal Circumference, Hip Circumference, WHtR (Waist/Height Ratio), with group 1 (first tercile) showing great reductions compared to the other two groups.

Related to the hemodynamic and health-related physical fitness variables, only the 6MWT showed significant difference between the first tercile and the third tercile (*p* < 0.01). In the group of biochemical parameters, there were significant differences between the first tercile against and the tercile in VLDL-c and TG, and also between the second tercile and the first one (*p* < 0.01).

Related to the indices derived from the biochemical and anthropometric parameters, Table 2 shows significant differences between the first tercile and the third tercile for AIP, TyG-BMI, and TyG-WC. Whereas the differences were also significant between the second and third terciles and the first and second terciles only for TYG-BMI (*p* < 0.01). 

## 4. Discussion

The main objective of this study was to analyze the responses promoted by an MTPO in adults, most of whom had severe obesity. In fact, 82% of the participants fell into this category, with an average BMI over 40 kg/m^2^ [30,41].

Considering the primary outcome, clinically significant weight loss, it was shown that at least one-third of the participants achieved a weight reduction over 5%, as illustrated in Figure 2. This is a notable outcome, especially since the MTPO was implemented under conditions similar to those found in public health services. The professionals involved in the intervention—psychologist, dietitian, and physical activity or exercise specialist—are commonly available in Brazil’s public health system. Using this type of strategy could make similar programs affordable enough to be broadly adopted within Brazil’s public health framework and possibly could be implemented in other developing countries too. Although program costs were not formally assessed, interventions delivered by non-medical professionals are generally less expensive than those led by general practitioners or nurses. Furthermore, the availability of existing institutional facilities helps to further reduce implementation costs. This suggests that the main challenge lies in formulating effective policies to facilitate the execution of such programs.

The results are consistent with findings in the literature regarding the proportion of participants who achieve clinically significant weight loss. Behavioral programs typically result in about 20% of participants meeting this target. In our study, over 25% achieved it [42]. More intensive interventions, generally conducted as RCTs under ideal conditions, tend to report higher success rates. For example, studies in Norway by Berge et al. and Gjevestad et al. demonstrated a higher proportion of participants reaching the 5% weight loss goal. In one such study, participants with higher cardiorespiratory fitness achieved an average weight loss of 7.6%, comparable to the average in the first tercile of our study. However, in the Norwegian study, this goal was achieved in just three months through a 6-hour-per-day program, three times per week for 12 weeks (216 h total). The sessions included two supervised training periods (60–90 min each), followed by lectures on nutrition, physical activity, and motivation. This highlights the significant role that intervention intensity plays and provides an important basis for comparison between efficacy and effectiveness trials [17,43]. In contrast, our study’s total intervention time was 96 h over 16 weeks (48 h of physical exercises and 48 h of theoretical classes). These classes addressed behavioral aspects such as eating habits, reducing sedentary behavior, and implementing sustainable increases in habitual physical activity.

It is very important to note that efficacy trials are typically developed under ideal conditions, requiring extensive infrastructure and personnel. Consequently, these programs are more intensive and often involve inpatient treatment, making them significantly more costly. Such approaches are usually feasible only in high-income countries.

Beyond weight loss, our study demonstrated that several other health parameters improved significantly—some even more than the weight loss itself. Notably, parameters related to glucose metabolism, such as insulin, HOMA-IR, and HOMA-Beta, showed improvements of approximately 20%. These findings highlight the multiple health benefits of such programs and the importance of addressing obesity-related comorbidities [15,44].

One challenge is that MTPO offered by hospitals and universities are less frequently accessed by the public than commercial programs. However, they often include components not commonly found in commercial programs [22].

While the weight loss outcomes met expectations, it is possible that some participants might not perceive the results as sufficient. Some studies suggest that a weight loss of over 30% is considered ideal, 25% acceptable, and anything below 17% disappointing [45]. Thus, educating participants on the clinical relevance of a 5% weight loss is critical for managing expectations and avoiding frustration when meaningful health benefits, like those seen in this study, are achieved [13]. Although our MTPO included both land-based and aquatic exercise programs, this variation likely had minimal impact. Both programs shared similar structures in terms of frequency (three times per week), intensity (moderate), and session duration (60 min). Additionally, participants were continuously encouraged to increase physical activity and reduce sedentary time.

Metabolic abnormalities are frequently observed in individuals with obesity [46], underscoring the importance of public health programs aimed at reducing these risks. While individual biomarkers are useful for health monitoring, composite indices such as the TyG index and AIP have been shown to be even stronger predictors of cardiovascular disease [47]. Our study demonstrated substantial improvements in both individual biochemical markers and these composite indices.

Obesity is closely linked with insulin resistance (IR), a relationship that was clearly evident in our study. Recently, TyG-related markers, which combine obesity indicators with the TyG index, have shown promise in evaluating IR in adults with obesity [48]. We assessed these markers as alternative indicators of IR, in line with current studies [48,49].

The findings not only revealed variations in these parameters but also showed their responsiveness to the MTPO. Interestingly, their response patterns mirrored those of the anthropometric measurements, underscoring the interconnectedness between obesity, IR, and the potential benefits of MTPOs.

Our results align with those of the Diabetes Prevention Program (DPP), which reported an average weight loss of around 7%. Even among participants in the other two terciles—who did not achieve substantial weight loss—there were still notable improvements in metabolic risk and physical fitness [50].

These findings emphasize the importance of evaluating not just weight loss but also other health improvements when assessing the effectiveness of obesity interventions [51]. A more comprehensive evaluation that includes various health parameters provides a holistic perspective on the overall benefits. Based on our experience with MTPO for adolescents, our group has also proposed a criterion for success that can be extended to adults [52].

A significant limitation of this study is the high dropout rate. Attrition is particularly problematic in weight management interventions, as participants who do not see progress are more likely to drop out. Studies show that trials with attrition rates above 20% tend to report less weight loss [26]. High dropout rates can reduce program effectiveness, compromise data integrity, and introduce bias into study results [53].

Despite this, attrition in weight management programs is a well-documented issue, with rates reaching up to 80% [54]. Our ~50% attrition rate is consistent with the literature, as many obesity intervention programs report similarly high dropout rates. Moreover, dropout tends to increase over time—ranging from 20–30% in the first 4 months to 85% after 36 months [54]. It is also important to highlight that the literature on attrition is highly heterogeneous, with ranges from 10% to 80% depending on the setting and the type of program [55].

Another limitation is the lack of a follow-up phase to assess the long-term sustainability of the results. Also, the absence of sensitivity analyses (e.g., intention-to-treat or imputation for missing data). Future studies should address this in order to better evaluate the lasting impact of such interventions. Moreover, a major strength of this study is that it was conducted in a developing country, where conducting such research is particularly challenging due to limited funding and resources. Additionally, this study was implemented in two different cities using the same protocol. This allowed us to generalize the results, despite differences such as exercise modality, and reflects the real-world variability professionals may face when implementing similar programs.

## 5. Conclusions

These results reinforce the evidence supporting the effectiveness of lifestyle intervention in promoting substantial weight loss and enhancements in cardiometabolic risk factors among a considerable proportion of individuals with obesity or severe obesity. They also confirm that pragmatic trials conducted in a developing country can yield meaningful outcomes, even for participants who did not achieve the clinically significant weight loss. These findings should be replicated in other developing countries under similar conditions, and future studies must include follow-up periods to assess the long-term sustainability of the results.

## Figures and Tables

**Table 1:** General effect of intervention comparing the intervention group (IG n = 180) with the control group (CG n = 30).

Anthropometric/Body Composition Variables	CG (n = 30)	f-Value	*p*-Value	np2	IG (n = 180)	f-Value	*p*-Value	np2	Mean Difference Between Groups	f-Value	*p*-Value	np2	Interaction
Sex T0	11 M 19 F	-	-	-	42 M 138 F	-	-	-	-	-	-	-	-
Sex T1	11 M 19 F				42 M 138 F								
Body Mass (kg) T0	114.27 ± 21.51	7.65	0.010 *	0.209	109.98 ± 23.46	74.04	0.000 *	0.293	2.00 (−5.20; 5.00)	36.22	0.000 *	0.152	0.007
Body Mass (kg) T1	116.70 ± 21.54				106.84 ± 23.01								
BMI (kg/m^2^) T0	40.94 ± 5.57	1.24	0.275	0.041	40.76 ± 6.90	79.31	0.000 *	0.307	0.77 (−2.10; 1.89)	20.26	0.000 *	0.018	0.026
BMI (kg/m^2^) T1	41.42 ± 5.74				39.57 ± 6.90								
Lean Body Mass (kg) T0	55.64 ± 9.79	0.16	0.693	0.005	48.46 ± 15.62	2.36	0.126	0.013	0.00 (−3.60; 1;40)	0.01	0.913	0.248	0.001
Lean Body Mass (kg) T1	55.39 ± 10.06				48.14 ± 15.12								
Skeletal Muscle Mass (kg) T0	32.89 ± 6.09	0.17	0.694	0.006	31.74 ± 6.40	0.25	0.234	0.028	0.20 (3.20; 1.10)	0.47	0.495	0.336	0.211
Skeletal Muscle Mass (kg) T1 T1(kg) T1	32.73 ± 6.21				31.70 ± 6.21								
Body fat (%) T0	47.89 ± 5.58	3.36	0.077	0.104	49.05 ± 5.84	31.65	0.000 *	0.150	0.80 (−3.00; 2.30)	10.06	0.002 *	0.192	0.045
Body fat (%) T1	48.54 ± 5.41				47.37 ± 6.00								
Absolute Body Fat (kg) T0	58.50 ± 14.28	0.86	0.361	0.029	61.54 ± 17.81	62.18	0.000 *	0.258	2.10 (−5.80; 4.50)	15.13	0.000 *	0.015	0.025
Absolute Body Fat (kg) T1	59.46 ± 14.44				58.71 ± 18.38								
Lean Mass/Fat Mass Ratio T0 (kg) T0	0.98 ± 0.23	11.14	0.002 *	0.278	0.84 ± 0.30	35.44	0.000 *	0.165	−0.03 (−0.30; 0.00)	5.89	0.016 *	0.154	0.133
Lean Mass/Fat Mass Ratio T1 (kg) T1	1.10 ± 0.26				0.90 ± 0.37								
Neck Circumference T0 (cm)T0	40.47 ± 4.86	0.16	0.693	0.005	39.83 ± 4.57	26.66	0.000 *	0.130	0.85 (−2.80; 2.00)	2.95	0.087	0.423	0.379
Neck Circumference (cm)T1 T1(cm) T1	40.34 ± 4.82				38.82 ± 4.23								
Waist Circumference (cm) T0	111.19 ± 12.48	4.39	0.045 *	0.131	109.72 ± 14.06	31.52	0.000 *	0.150	2.50 (−11.20; 7.10)	13.47	0.000 *	0.155	0.011
Waist Circumference (cm)T1	113.65 ± 13.80				106.04 ± 13.75								
Hip Circumference (cm) T0	127.02 ± 13.62	2.54	0.122	0.081	127.15 ± 13.59	12.71	0.000 *	0.066	1.40 (−11.00; 6.00)	5.85	0.016 *	0.043	0.043
Hip Circumference (cm) T1	129.01 ± 13.85				124.41 ± 41.55								
Waist/Height Ratio (cm)T0	0.66 ± 0.64	4.56	0.041 *	0.136	0.66 ± 0.07	28.94	0.000 *	0.139	0.01 (−0.06; 0.04)	12.69	0.000 *	0.039	0.030
Waist/Height Ratio (cm)T1	0.68 ± 0.75				0.64 ± 0.07								
Hemodynamic/Health-Related Physical Fitness Variables	GC (n = 30)	f-Value	*p*-Value	np2	IG (n = 180)	f-Value	*p*-Value	np2	Mean Difference between Groups	f-Value	*p*-Value	np2	Interaction
Systolic Blood Pressure (mmHg) T0	125.94 ± 9.98	1.29	0.265	0.043	126.28 ± 11.30	25.69	0.000 *	0.125	4.00 (−16.66; 11.33)	0.50	0.481	0.086	0.018
Systolic Blood Pressure (mmHg) T1	123.07 ± 11.45				121.69 ± 11.28								
Diastolic Blood Pressure (mmHg)T0	78.83 ± 8.79	3.38	0.076	0.104	81.21 ± 10.60	15.79	0.000 *	0.081	2.66 (−20.00; 9.66)	11.40	0.001 *	0.041	2.537
Diastolic Blood Pressure (mmHg) T1	83.04 ± 8.19				77.96 ± 8.11								
HR (bpm) T0	81.23 ± 10.08	1.80	0.191	0.058	81.48 ± 12.21	1.48	0.225	0.008	2 (−23; 9)	0.31	0.581	0.008	0.075
HR (bpm) T1	78.43 ± 9.03				80.19 ± 13.55								
Distance 6MWT (m) T0	502.61 ± 49.43	6.68	0.015 *	0.187	503.07 ± 73.55	0.27	0.603	0.002	−8.50 (−119.40; 42.50)	3.73	0.055	0.023	0.055
Distance 6MWT (m) T1	473.92 ± 73.97				506.48 ± 85.01								
Static Abdominal Muscle Endurance (s) T0	39.51 ± 32.84	1.04	0.317	0.035	29.53 ± 25.75	161.33	0.000 *	0.474	15.00 (−19.00; 30.00)	8.28	0.004 *	0.140	0.011
Static Abdominal Muscle Endurance (s) T1	33.52 ± 28.33				49.64 ± 31.67								
Dynamic Lower Limb Muscular Endurance (n rep.) T0	12.77 ± 2.23	0.60	0.446	0.020	13.66 ± 3.94	181.33	0.000 *	0.503	−2 (−7; −1)	16.79	0.000 *	0.144	0.314
Dynamic Lower Limb Muscular Endurance (n rep.) T1	13.23 ± 2.96				16.66 ± 3.92								
Flexibility (cm) T0	14.87 ± 6.74	1.20	0.282	0.040	17.45 ± 10.14	39.93	0.000 *	0.182	−2.66 (−14.00; 0.00)	2.84	0.094	0.161	0.030
Flexibility (cm) T1	15.99 ± 8.21				20.99 ± 8.81								
Biochemical Parameters	CG	f-Value	*p*-Value	np2	IG (n = 180)	f-Value	*p*-Value	np2	Mean Difference between Groups	f-Value	*p*-Value	np2	Interaction
Glycemia (mg/dL) T0	106.87 ± 43.97	0.04	0.848	0.001	105.94 ± 37.05	11.21	0.001 *	0.059	4 (−15; 11)	1.04	0.310	0.017	0.203
Glycemia (mg/dL) T1	105.73 ± 23.74				99.29 ± 36.22								
Insulin (mU/L) T0	24.86 ± 12.20	0.83	0.370	0.028	22.03 ± 11.40	78.51	0.000 *	0.305	4.41 (−5.37; 9.78)	6.34	0.013 *	0.104	0.100
Insulin (mU/L) T1	23.33 ± 14.69				15.88 ± 9.01								
Homa IR T0	6.89 ± 5.84	0.42	0.524	0.014	5.82 ± 4.15	71.24	0.000 *	0.285	1.21 (−1.81; 2.62)	4.15	0.043 *	0.108	0.001
Homa IR T1	6.31 ± 5.39				3.89 ± 2.53								
Homa β T0	84.60 ± 42.41	2.27	0.143	0.072	75.54 ± 41.15	51.90	0.000 *	0.225	13.83 (−25.12; 35.99)	2.75	0.099	0.081	0.029
Homa β T1	76.58 ± 44.78				55.86 ± 31.62								
hs-CRP (mg/L) T0	8.60 ± 10.14	0.82	0.372	0.028	5.83 ± 5.76	0.01	0.904	0.000	0.08 (−6.75; 2.26)	1.44	0.232	0.032	0.011
hs-CRP (mg/L) T1	7.30 ± 7.36				5.78 ± 5.76								
Total Cholesterol (mg/dL) T0	192.21 ± 28.99	2.58	0.119	0.082	194.04 ± 37.32	6.74	0.010 *	0.036	2.70 (−41.40; 16.00)	5.73	0.018 *	0.013	0.135
Total Cholesterol (mg/dL) T1	200.67 ± 29.66				188.07 ± 33.99								
HDL-c (mg/dL) T0	46.90 ± 13.36	0.48	0.494	0.016	47.34 ± 11.78	3.59	0.060 *	0.020	0 (−21; 5)	1.47	0.227	0.130	1.323
HDL-c (mg/dL) T1	45.97 ± 11.29				48.81 ± 13.75								
LDL-c (mg/dL) T0	117.25 ± 29.24	1.95	0.173	0.063	117.84 ± 33.08	6.87	0.010 *	0.037	1.90 (−38.40; 17.00)	4.51	0.035 *	0.014	0.012
LDL-c (mg/dL)T1	123.60 ± 29.92				112.03 ± 31.28								
VLDL-c (mg/dL) T0	30.96 ± 14.22	1.48	0.233	0.049	28.15 ± 15.71	6.92	0.009 *	0.037	1.20 (−14.00; 7.00)	0.06	0.811	0.058	0.311
VLDL-c (mg/dL)T1	27.87 ± 13.78				25.64 ± 12.28								
Non-HDL Cholesterol (mg/dL) T0	147.84 ± 30.21	2.04	0.164	0.066	136.68 ± 44.67	0.47	0.494	0.003	−2.20 (−79.00; 13.00)	0.42	0.515	0.051	0.189
Non-HDL Cholesterol (mg/dL)T1	155.02 ± 31.50				138.68 ± 31.91								
Triglycerides (mg/dL) T0	160.63 ± 91.33	0.01	0.943	0.000	147.20 ± 95.43	12.14	0.001 *	0.064	6 (−70; 39)	1.83	0.178	0.062	0.364
Triglycerides (mg/dL) T1	161.77 ± 120.49				128.98 ± 65.91								
Glycated Hemoglobin (%) T0	5.59 ± 1.11	1.08	0.307	0.036	5.69 ± 1.18	13.97	0.000 *	0.072	0 (−0.60; 0.20)	4.75	0.030 *	0.007	0.349
Glycated Hemoglobin (%) T1	5.73 ± 0.62				5.50 ± 1.09								
Indices Derived From Biochemical/Anthropometric Parameters													
AIP (mg/dL) T0	4.06 ± 3.73	0.09	0.766	0.003	3.50 ± 3.11	8.91	0.003 *	0.047	0.13 (−1.81; 0.88)	2.02	0.156	0.096	1.019
AIP (mg/dL) T1	4.22 ± 4.40				2.99 ± 2.43								
MetS–Z BMI T0	1.09 ± 0.89	0.33	0.572	0.011	1.07 ± 1.07	25.35	0.000 *	0.124	0.23 (−0.59; 0.47)	3.19	0.076	0.028	0.059
MetS–Z BMI T1	1.02 ± 1.06				0.79 ± 1.01								
Percentil BMI T0	79.65 ± 14.08	0.40	0.534	0.013	77.95 ± 17.30	50.46	0.000 *	0.220	4.58 (−13.63; 12.29)	4.89	0.028 *	0.045	0.006
Percentil BMI T1	78.33 ± 19.14				71.03 ± 21.12								
MetS-Z WC T0	0.83 ± 0.92	0.21	0.653	0.007	0.76 ± 1.02	26.67	0.000 *	0.130	0.22 (−0.63; 0.52)	7.32	0.007 *	0.047	0.169
MetS-Z WC T1	0.87 ± 0.91				0.47 ± 0.97								
Percentil WC T0	72.40 ± 17.41	0.14	0.710	0.005	70.65 ± 20.18	45.97	0.000 *	0.204	4.67 (−18.11; 14.08)	8.40	0.004 *	0.074	0.031
Percentil WC T1	73.31 ± 21.18				62.81 ± 22.68								
TYG (mg/dL) T0	4.79 ± 0.31	0.25	0.623	0.008	4.73 ± 0.31	20.47	0.000 *	0.103	0.04 (−0.29; 0.19)	1.23	0.269	0.064	0.000
TYG (mg/dL) T1	4.77 ± 0.34				4.66 ± 0.26								
TYG-BMI T0	364.86 ± 58.46	0.30	0.589	0.010	358.25 ± 68.05	76.49	0.000 *	0.299	11.32 (−27.22; 27.24)	14.51	0.000 *	0.041	0.048
TYG-BMI T1	367.46 ± 62.80				341.97 ± 65.53								
TYG-WC T0	991.98 ± 150.95	1.26	0.270	0.042	964.88 ± 154.70	45.28	0.000 *	0.202	31.93 (−111.56; 86.90)	12.28	0.001 *	0.175	0.015
TYG-WC T1	1008.33 ± 157.94				918.36 ± 139.57								

ANOVA two-way GLM; *—significant difference; HR—heart rate; bpm—beats per minute; mg/dL—milligrams per deciliter; hs-CRP—high-sensitivity C-reactive protein; HDL—high-density; WC—waist circumference. Lipoprotein; LDL—low-density lipoprotein; AIP—atherogenic index of plasma; BMI—Body Mass Index; TYG—Triglyceride-Glucose Index; protein; HDL—high-density lipoprotein; LDL—low-density lipoprotein; AIP—atherogenic index of plasma; BMI—Body Mass Index; TYG—Triglyceride-Glucose Index; WC—Waist Circumference.

**Table 2:** Relative (%) responses in the intervention group (IG) stratified by the percentual of weight loss in terciles (n = 180).

Anthropometric/Body Composition Variables	Tercil 1 Responders (n = 60)	Tercil 2 (n = 60)	Tercil 3 (n = 60)	f-Value	*p*-Value	np2
Sex	13 M 47 F	19M 41F	10M 50F	-	-	-
Age (years)	40.38 ± 8.50	39.60 ± 8.922	37.82 ± 8.99	1.337	0.265	0.015
Height (cm)	165.34 ± 8.21	164.25 ± 10.04	162.78 ± 9.74	1.126	0.327	0.013
Body Mass (%)	−7.61 ± 3.74 ^a,b^	−2.37 ± 0.81 ^a,c^	1.49 ± 1.77 ^b,c^	210.612	0.000 *	0.704
Body Mass Index (%)	−7.57 ± 3.76 ^a,b^	−2.43 ± 0.97 ^a,c^	1.29 ± 2.17 ^b,c^	179.716	0.000 *	0.670
Lean Body Mass (%)	−1.45 ± 5.83	0.30 ± 9.07	0.70 ± 13.20	0.822	0.441	0.009
Skeletal Muscle Mass (%)	−3.48 ± 10.67 ^b^	−1.77 ± 8.44	1.50 ± 12.95 ^b^	3.284	0.040 *	0.036
Body Fat (%)	−6.28 ± 7.73 ^a,b^	−1.96 ± 9.53 ^a,c^	−1.54 ± 6.19 ^b,c^	6.573	0.002 *	0.069
Absolute Body Fat (%)	−12.71 ± 8.43 ^a,b^	−3.95 ± 3.92 ^a,c^	1.68 ± 4.54 ^b,c^	88.261	0.000 *	0.499
Lean Mass/Fat Mass Ratio (%)	14.18 ± 15.60 ^a,b^	4.88 ± 12.91 ^a^	−0.37 ± 16.35 ^b^	14.429	0.000 *	0.140
Neck Circumference (%)	−3.08 ± 5.90	−2.14 ± 7.07	−1.29 ± 6.03	1.192	0.306	0.013
Waist Circumference (%)	−5.72 ± 7.14 ^b^	−3.64 ± 6.62 ^c^	0.23 ± 8.93 ^b,c^	9.420	0.000 *	0.096
Hip Circumference (%)	−5.21 ± 7.51 ^b^	−1.93 ± 8.54	1.37 ± 7.16 ^b^	10.785	0.000 *	0.109
Waist/Height Ratio (%)	−5.72 ± 7.14 ^b^	−3.64 ± 6.62 ^c^	0.23 ± 8.93 ^b,c^	9.420	0.000 *	0.096
Hemodynamic/Health-Related Physical Fitness Variables	Tercil 1 Responders (n = 60)	Tercil 2 (n = 60)	Tercil 3 (n = 60)	f-Value	*p*-Value	np2
Systolic Blood Pressure (%)	−4.51 ± 8.68	−2.39 ± 10.44	−2.72 ± 9.31	0.864	0.423	0.010
Diastolic Blood Pressure (%)	−5.74 ± 12.03	−0.15 ± 21.67	−1.79 ± 14.29	1.988	0.140	0.022
HR (%)	−4.44 ± 16.86	0.36 ± 18.89	3.02 ± 16.69	2.809	0.063	0.031
Distance 6MWT (%)	7.24 ± 18.06 ^b^	−1.08 ± 15.96	−0.77 ± 15.48 ^b^	4.890	0.009 *	0.052
Static Abdominal Muscle Endurance (%)	590.87 ± 1499.17	534.03 ± 1200.43	320.21 ± 903.15	0.814	0.445	0.009
Dynamic Lower Limb Muscle Endurance (%)	23.29 ± 20.85	34.78 ± 72.74	17.47 ± 18.42	2.300	0.103	0.025
Flexibility (%)	93.90 ± 284.77	77.87 ± 228.75	31.30 ± 103.75	1.320	0.270	0.015
Biochemical Parameters	Tercil 1 Responders (n = 60)	Tercil 2 (n = 60)	Tercil 3 (n = 60)	f-Value	*p*-Value	np2
Glycemia (%)	−9.91 ± 9.33	−10.36 ± 11.91	−9.75 ± 9.38	2.687	0.071	0.029
Insulin (%)	−29.95 ± 18.51	−24.33 ± 22.19	−33.40 ± 23.34	0.318	0.728	0.004
Homa IR (%)	−32.22 ± 20.77	−29.20 ± 24.68	−35.72 ± 25.73	0.108	0.898	0.001
Homa β (%)	−23.36 ± 25.36	−11.53 ± 36.03	−26.05 ± 32.19	1.481	0.230	0.016
hs-CRP (%)	−36.21 ± 25.43	−27.23 ± 24.44	−33.99 ± 30.71	2.567	0.080	0.028
Total Cholesterol (%)	−2.93 ± 15.78	−1.44 ± 12.47	−1.00 ± 15.19	0.290	0.749	0.003
HDL-c (%)	4.22 ± 19.55	6.16 ± 18.85	2.52 ± 26.35	0.418	0.659	0.005
LDL-c (%)	2.89 ± 28.35	−4.26 ± 24.21	−4.54 ± 20.00	1.786	0.171	0.020
VLDL-c (%)	−11.15 ± 30.44 ^b^	4.96 ± 34.43	8.19 ± 50.73 ^b^	4.125	0.005 *	0.045
Non-HDL Cholesterol (%)	0.11 ± 28.11 ^b^	16.89 ± 63.11	32.87 ± 106.56 ^b^	2.996	0.053	0.033
Triglycerides (%)	−13.90 ± 29.97 ^a,b^	4.45 ± 34.85 ^a^	3.98 ± 37.92 ^b^	5.554	0.005 *	0.059
Glycated Hemoglobin (%)	−1.67 ± 9.06	−1.53 ± 6.97	−0.24 ± 6.00	0.661	0.517	0.007
Indices Derived from Biochemical/ Anthropometric Parameters						
AIP (%)	−13.41 ± 36.91 ^b^	0.80 ± 35.20	5.90 ± 45.41 ^b^	3.871	0.023 *	0.042
MetS–Z BMI (%)	−72.96 ± 349.50	−63.38 ± 335.25	25.14 ± 251.98	1.767	0.174	0.020
Percentil BMI (%)	−12.56 ± 18.65	−8.70 ± 19.79	−5.84 ± 17.62	1.947	0.146	0.022
MetS-Z WC (%)	−84.81 ± 411.16	−69.80 ± 497.94	27.68 ± 243.59	0.098	0.907	0.001
Percentil WC (%)	−12.56 ± 18.65	−8.70 ± 19.79	−5.84 ± 17.62	1.846	0.161	0.020
TYG (%)	−2.29 ± 4.58	−1.01 ± 4.49	−0.79 ± 4.17	1.995	0.139	0.022
TYG-BMI (%)	−9.97 ± 5.78 ^a,b^	−3.48 ± 4.92 ^a,c^	0.43 ± 5.16 ^b,c^	58.969	0.000 *	0.400
TYG-WC (%)	−7.44 ± 8.55 ^b^	−4.64 ± 8.55	−0.58 ± 10.30 ^b^	8.478	0.000 *	0.087

^a^ Tercil 1 x 2; ^b^ Tercil 1 x 3; ^c^ Tercil 2 x 3 Post Hoc Bonferroni; *—significant difference; HR—heart rate; bpm—beats per minute; mg/dL—milligrams per deciliter; hs-CRP—high-sensitive C-reactive protein; HDL—high-density lipoprotein; LDL—low-density lipoprotein; AIP—atherogenic index of plasma; BMI—Body Mass Index; TYG—Triglyceride-Glucose Index; WC—Waist Circumference.

## Data Availability

The data presented in this study are available upon reasonable request from the corresponding author. The data are not publicly available due to privacy and ethical restrictions.

## Abbreviations

The following abbreviations are used in this manuscript:

MTPO	multidisciplinary treatment program of obesity
BMI	Body mass index
LM/FM	lean mass to fat mass ratio
HOMA-IR	homeostatic model assessment for insulin resistance
HOMA-B	homeostatic model assessment for beta
TG	triglycerides
H1Ac	hemoglobin A1C
PCT	pragmatic clinical trial
RCT	randomized controlled trials
WC	waist circumference
BP	blood pressure
IG	intervention group
CG	control group
DBP	diastolic blood pressure
LDL-c	low-density lipoprotein cholesterol
MetS-Z WC	metabolic syndrome severity z-score waist circumference
TyG	Triglyceride-Glucose Index
SBP	systolic blood pressure
6MWT	6-Minute Walk Test
VLDL-c	very-low-density lipoprotein cholesterol
AIP	atherogenic index of plasma
